# Vertebrate Fidgetin Restrains Axonal Growth by Severing Labile Domains of Microtubules

**DOI:** 10.1016/j.celrep.2015.08.017

**Published:** 2015-09-03

**Authors:** Lanfranco Leo, Wenqian Yu, Mitchell D’Rozario, Edward A. Waddell, Daniel R. Marenda, Michelle A. Baird, Michael W. Davidson, Bin Zhou, Bingro Wu, Lisa Baker, David J. Sharp, Peter W. Baas

**Affiliations:** 1 Department of Neurobiology and Anatomy, Drexel University College of Medicine, Philadelphia, PA 19129, USA; 2 Department of Biology, Drexel University, Philadelphia, PA 19104, USA; 3 National High Magnetic Field Laboratory and Department of Biological Science, Florida State University, Tallahassee, FL 32310, USA; 4 Department of Genetics, Albert Einstein College of Medicine, Bronx, NY 10461, USA; 5 Department of Physiology and Biophysics, Albert Einstein College of Medicine, Bronx, NY 10461, USA

## Abstract

Individual microtubules (MTs) in the axon consist of a stable domain that is highly acetylated and a labile domain that is not. Traditional MT-severing proteins preferentially cut the MT in the stable domain. In *Drosophila*, fidgetin behaves in this fashion, with targeted knockdown resulting in neurons with a higher fraction of acetylated (stable) MT mass in their axons. Conversely, in a fidgetin knockout mouse, the fraction of MT mass that is acetylated is lower than in the control animal. When fidgetin is depleted from cultured rodent neurons, there is a 62% increase in axonal MT mass, all of which is labile. Concomitantly, there are more minor processes and a longer axon. Together with experimental data showing that vertebrate fidgetin targets unacetylated tubulin, these results indicate that vertebrate fidgetin (unlike its fly ortholog) regulates neuronal development by tamping back the expansion of the labile domains of MTs.

## Graphical Abstract

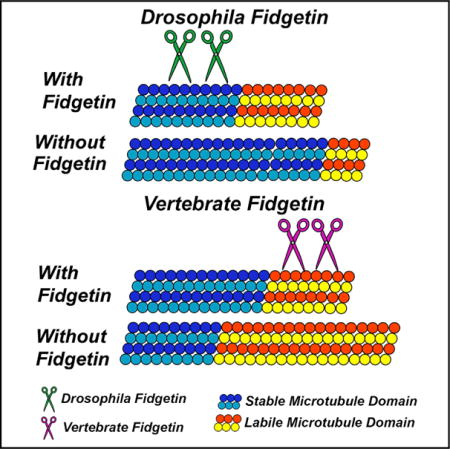

## INTRODUCTION

In the axon, each microtubule (MT) consists of a stable domain from which can elongate a labile domain (Baas and Black, 1990). The stable and labile domains are situated toward the minus and plus ends of the MT, respectively, with the labile portion of the MT directed toward the tip of the axon. For axons to grow in a robust fashion, there must be an expansion of the MT array, and especially of the labile domains. Also important for axonal growth is a process whereby long MTs are severed into shorter ones by proteins that tug at the MT lattice to induce breakage (Roll-Mecak and Vale, 2008). If the breakage occurs in the stable domain of an MT, the result is two new MTs, both capable of growing longer. This allows for an increase in MT number, locally within the axon, for example, at sites of branch formation (Qiang et al., 2010). If this breakage were to occur in a labile domain, the expected result would be just one MT that is shorter than the original, as an MT with no stable domain would depolymerize completely. Traditional MT-severing proteins, katanin and spastin, preferentially sever MTs in the stable domain (Riano et al., 2009). In theory, an MT-severing protein that targets the labile domain could be important for limiting the expansion of the MT array of the axon to modulate its growth. While *Drosophila* fidgetin (Fgn) is a traditional MT-severing protein (Zhang et al., 2007), its vertebrate ortholog diverges in sequence and structure (Yang et al., 2006), and yet is still able to sever MTs (Mukherjee et al., 2012). Results of our study indicate that vertebrate Fgn regulates neuronal development by targeting labile domains of MTs.

## RESULTS

### Fgn Depletion Affects Axonal Development and MTs in *Drosophila*

**Figure 1:**
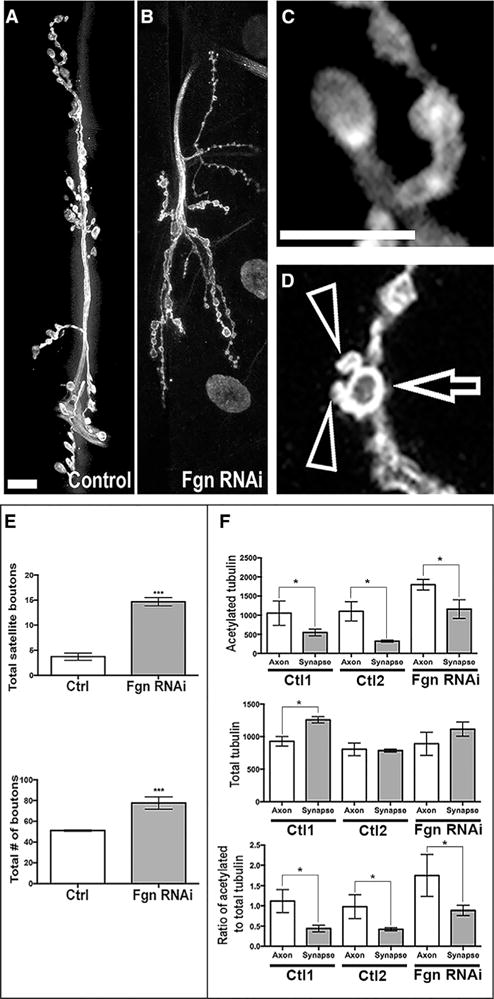
*Drosophila* Fgn Knockdown Increases Synaptic Connections In Vivo (A–D) Confocal images of third instar larval NMJs, muscles 6 and 7, labeled with α-HRP (white) to detect presynaptic neuronal membranes. The following are shown: (A) a Ctl NMJ; (B) an NMJ from an Fgn-knockdown animal (*Dcr2;UAS:Fgn*-*RNAi;elav*-*Gal4*; note the significant increase in bouton number in Fgn-knockdown animals compared to Ctls); (C) a representative individual bouton from Ctl animals; (D) a bouton with associated satellite boutons from an Fgn-knockdown animal. Arrows show the bouton; arrowheads show satellites. Scale bar represents 10 μm. (E) Quantification of the number of total boutons and satellite boutons in Fgn-knockdown animals compared to Ctls is shown. (F) Quantification of IF intensity of acetylated tubulin (top), total tubulin (middle), and ratio of acetylated to total tubulin (bottom) between Fgn-knockdown animals (*Dcr2;UAS:Fgn*-*RNAi;elav*-*Gal4*) and outcrossed Ctls (*Dcr2;;elav*-*Gal4* and *UAS:Fgn*-*RNAi*/+) at both the axonal shaft and distal synapse near the bouton. Note the significant increase of the ratio of acetylated tubulin to total tubulin. Error bars represent SE. *p < 0.05, ***p < 0.001.

The effects of knocking down *Drosophila* Fgn from fly neurons in the neuromuscular junction (NMJ) were investigated first. The Gal4/UAS system (Brand and Perrimon, 1993) was used to express an RNAi hairpin targeting the *Drosophila* Fgn gene (CG3326, or *Fgn*) under UAS control (*UAS:Fgn*-*RNAi*). Expression was limited to post-mitotic neurons using the *Elav*-*gal4* driver (Campos et al., 1987). The total number of synaptic contacts (boutons) was significantly higher in Fgn-knockdown animals (Figures 1B and 1E) compared to outcrossed control (Ctl) animals (Figures 1A and 1E). The number of satellite boutons, which are small growths of presynaptic membranes that extend out from axonal terminal arbors, in Fgn*-*knockdown animals (arrows in Figures 1D and 1E) was greater than that of Ctls (Figures 1C and 1E). An increased number of satellite boutons has been observed in flies bearing mutations to other MT-severing proteins (Sherwood et al., 2004; Mao et al., 2014). A significant increase in the ratio of acetylated to total tubulin (measured by quantifying fluorescence intensity) was observed in the axonal shaft and distal synapse, compared to two independent control RNAi lines (Ctl1 and Ctl2) (Figure 1F). This was due to a significant increase in MT acetylation in these areas, as total tubulin levels remained relatively unchanged (Figure 1F). A similar result has been reported in *Drosophila* neurons with compromised katanin (Mao et al., 2014). These results are consistent with *Drosophila* Fgn behaving similarly to traditional MT-severing proteins in the neurons of the fly.

### Fgn Expression in Developing Neurons

**Figure 2:**
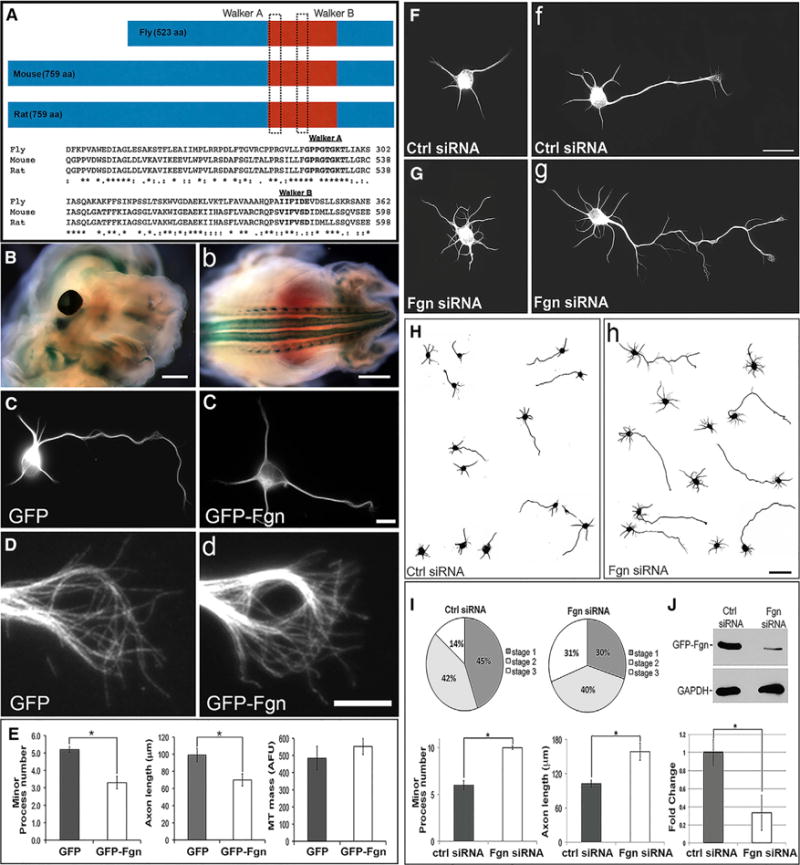
Studies on Vertebrate Fgn Expression in Rodent Neurons (A) Amino acid (aa) alignment of Fgn orthologs from rat (759 aa), mouse (759 aa), and *Drosophila* (523 aa). The two vertebrate orthologs are 99% identical, but notably different from *Drosophila* Fgn (see Results). (B) X-gal staining of Fgn knockout/reporter mouse fetus at E12.5. Fgn is highly expressed in CNS regions, such as brain and eye (left) and spinal cord (right). (C–E)MT immunostaining (anti-βIII-tubulin antibody) in cortical neurons expressing either GFP or GFP-Fgn. Fgn-overexpressing neurons have statistically shorter axons (70.04 ± 6.95 μm) and fewer minor processes (3.33 ± 0.163) compared to GFP-expressing Ctl neurons (axon length, 99.44 ± 8.0 μm; minor process number, 5.22 ± 0.35). Quantification is shown in (E) (Student’s t test, p ≤ 0.05). No difference in growth cone morphology or relative MT mass was observed as a result of Fgn overexpression (D, left and right). Similarly, there was no significant difference in MT mass in the axon (Student’s t test, p ≥ 0.05; E, third graph from left) between Ctl GFP (484 ± 69.42 AFU) and Fgn-expressing neurons (552 ± 46.95). Scale bars represent 0.5 mm (B, left), 2 mm (B, right), 10 μm (C, right), and 5 μm (D, right). (F–J) The effect on cortical neurons of Fgn siRNA pool. (F–H) Immunostains for βIII-tubulin. (F, left) and (G, left) are stage 2 neurons from Ctl and Fgn siRNA groups, respectively, while (F, right) and (G, right) are stage 3 neurons from the same experimental groups. Fgn depletion increases axon length (158.59 ± 14.90 μm; G, right and I, right), minor process number (10 ± 0.183; G, left and right and I, left), and rate of polarization (stage 1, 30% ± 5.6%; stage 2, 40% ± 3.5%; stage 3, 31% ± 2.5% (H, left and I, left) compared to Ctl siRNA neurons (F, left and right and H, left; axon length, 102.77 ± 5.95 μm; process number, 6 ± 0.47; polarization stage 1, 45% ± 4.4%; stage 2, 42% ± 3.5%; stage 3, 14% ± 1.7%) (Student’s t test p ≤ 0.05). Quantification is shown in (I). Validation of Fgn siRNA is shown in (J) where RFL-6 cells were co-transfected with GFP-Fgn and either Ctl siRNA (left) or Fgn siRNA (right). Fgn siRNA reduced (Student’s t test, p ≥ 0.05) by more than 70% the GFP-Fgn expression observed with Ctl siRNA, as evaluated by western blotting (fold change: Ctl siRNA, 1 ± 0.104; Fgn siRNA, 0.28 ± 0.194; J and immunocytochemistry [data not shown]). Scale bars represent 20 μm (F, right) and 50 μm (H, right).

Fgn was discovered in vertebrates as a gene spontaneously mutated in a mouse strain that displayed a fidgeting phenotype (Cox et al., 2000). As shown in Figure 2A, vertebrate Fgn is larger than *Drosophila* Fgn, with a region of over 300 amino acids toward the N terminus that is absent from the fly ortholog. The Walker A motif in the AAA region is the same as in fly, but the Walker B has unusual amino acid substitutions. Multiple attempts at developing Fgn antibodies in the past have failed for unknown reasons (Yang et al., 2005). Here, a line of mice that knocks out Fgn by replacing most of the Fgn gene for LacZ was purchased, so that Fgn’s expression pattern could be observed by staining for β-galactosidase. Fgn expression was observed in various tissues, but was especially high in developing nervous tissue (Figure 2B).

Like cultures of fetal rat hippocampal neurons used in relevant previous studies (Qiang et al., 2010), cortical neurons undergo stereotyped developmental stages in which a lamellipodium (stage 1) becomes multiple minor processes (stage 2), one of which then becomes the axon (stage 3) after which the rest become dendrites (stage 4). Consistent with previous studies with mouse GFP-Fgn (Yang et al., 2005), ectopically expressed rat GFP-Fgn was found to reside in the nucleus, but was also cytoplasmic, distributing throughout the neuron. The morphological effects of Fgn overexpression were a shorter axon and fewer immature processes (Figure 2C and quantification in Figure 2E). There was no evidence of short fragmented MTs as a result of Fgn expression. For example, in Figure 2D, long MTs appear in the growth cone in GFP-Fgn-expressing neurons as well as Ctl GFP-expressing neurons, without any obvious short MT fragments. Per unit length of axon, there was no difference in MT levels in Ctl and Fgn-depleted axons (Figure 2E). Whether the construct was GFP-Fgn or Fgn-GFP or whether a flag tag was used, no fragmentation of MTs was observed in neurons or in rat fibroblasts (Figure S1).

### Effects of Fgn Depletion on Cultured Vertebrate Neurons

Small interfering RNA (siRNA) was introduced just prior to plating. After 2 days of protein depletion, dense cultures were re-plated at a lower density to quantify differences in neuronal morphology when processes were permitted to grow a new. A day after re-plating, the total number of minor processes per cell body was roughly doubled in Fgn-depleted neurons (Figure 2G) compared to Ctl (Figure 2F), and Fgn-depleted neurons had significantly longer axons (see also Figure 2H, which shows tracings of additional Ctl and Fgn-depleted neurons). Differentiation was accelerated as a result of Fgn depletion, with Fgn-depleted cultures at 24 hr having a significantly higher percentage of neurons in stage 3 compared to Ctl siRNA, and a corresponding reduction in the percentage of neurons in stage 1 was observed. Morphological effects of Fgn depletion were essentially the inverse of the effects resulting from overexpression. Data are shown in Figure 2I For confirmation of knockdown, GFP-Fgn was expressed in fibroblasts or neurons together with the siRNA for 24 hr. In western blot analyses, the GFP-Fgn band, detected with a GFP antibody, was reduced by over 70% in the cultures transfected with Fgn siRNA compared to those transfected with Ctl siRNA (Figure 2J). Similar results were obtained on cultures in which each of the siRNA sequences was used individually (Figure S2).

### Fgn Depletion Increases Labile MT Mass in Axons

**Figure 3:**
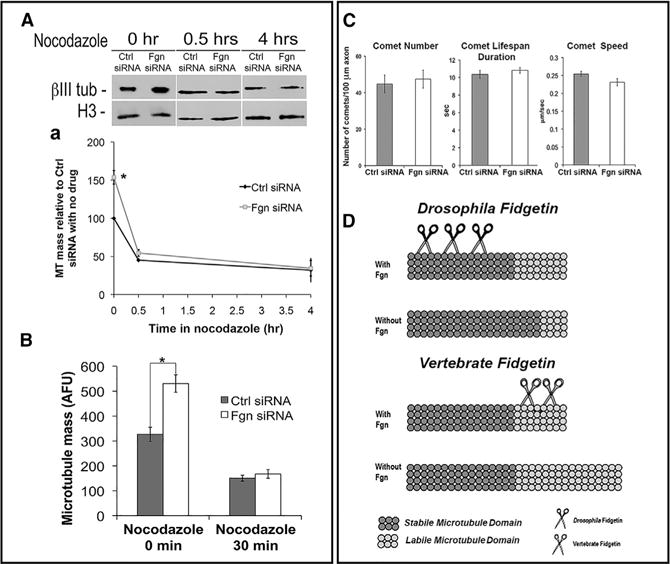
Vertebrate Fgn Depletion Increases Labile MT Mass in the Axon but Does Not Alter MT Number (A) Cortical neurons that had been treated for 48 hr with Fgn or Ctl siRNA and then re-plated were treated with nocodazole (0.2 μg/ml) for 0 min, 30 min, or 4 hr. Cultures were pre-extracted to release free tubulin and then prepared for IF or western blotting. Fgn-depleted cultures had 52.75% ± 8.9% greater MT mass compared to Ctl siRNA (one-way ANOVA, p ≤ 0.05). At 30-min nodocazole, MT mass decreased to roughly half (oneway ANOVA, p ≤ 0.05) compared to the nocodazole-free time point (Ctl siRNA, 45.21% ± 1.21% decrease; Fgn siRNA, 54.71% ± 15.92% decrease), with no difference in MT levels between the Ctl siRNA and Fgn siRNA groups (one-way ANOVA, p ≥ 0.05). Fgn-depleted cultures and Ctl cultures also did not differ in MT mass (one-way ANOVA, p ≥ 0.05) after 4 hr in nocodazole (Ctl siRNA, 31.97% ± 5.46%; Fgn siRNA, 34.78% ± 10.27%). Quantification of MT mass in the axon was by IF. (B) Fgn depletion increases the overall MT mass in the axon by 62% (Ctl siRNA, 327.09 ± 28.26; Fgn siRNA, 530.55 ± 34.88; one-way ANOVA, p ≤ 0.05). No differences were observed (one-way ANOVA, p ≥ 0.05) between Fgn siRNA and Ctl siRNA after treatment with nocodazole (0.2 μg/ml) for 30 min (Ctl siRNA, 150.34 ± 11.84; Fgn siRNA, 167.36 ± 17.66). (C) Neurons were transfected at day 0 with Fgn or Ctl siRNA and re-transfected with EB3-GFP at the time of re-plating, so that dynamic plus ends of MTs could be visualized as fluorescent comets (tracked as 1 frame/s) during bouts of MT assembly. The two experimental groups did not differ (Student’s t test, p ≥ 0.05) in terms of comet number (Ctl siRNA, 44.70 ± 14.43; Fgn siRNA, 47.40 ± 14.39), duration (Ctl siRNA, 10.35 ± 0.46 s; Fgn siRNA, 10.82 ± 0.33 s), or speed (Ctl siRNA, 0.25 ± 0.0073 μm/s; Fgn siRNA, 0.23 ± 0.0098 μm/s). (D) Schematic shows interpretation of data. *Drosophila* Fgn behaves similarly to spastin or katanin, targeting the stable domain of axonal MTs. Knockdown causes an increase in the proportion of the MT that is stable, without altering the length of the MT. Vertebrate Fgn targets the labile domain of axonal MTs, such that knocking down vertebrate Fgn increases the length of the labile domain.

In neurons depleted of Fgn, there was a 53% ± 8.9% increase in MT mass relative to Ctls as assessed by western blotting (Figure 3A), and a 62% ± 8.63% increase in MT mass per unit area of axon as assessed by quantitative immunofluorescence (IF) (Figure 3B). Nocodazole sensitivity was used to discern the stable and labile fractions of the MT mass (Baas and Black, 1990). After 30 min of drug treatment, the MT levels were indistinguishable from the corresponding drug-treated Ctls, as assessed by either western blotting or IF (Figures 3C and 3D), indicating that the MT mass added as a result of Fgn depletion is entirely labile.

GFP-EB3 (which tracks dynamic plus ends of MTs) was expressed at the time of re-plating in neurons that had been transfected 2 days earlier with either Fgn or Ctl siRNA. No difference in the number of the GFP-EB3 comets was found between Fgn-depleted and Ctl axons, nor were there any differences in the rate or duration of the comets (Figure 3C). These results, in conjunction with the results of the nocodazole study, suggest that Fgn depletion does not increase MT number and, therefore, must lengthen the labile domains of MTs (Figure 3D). By contrast, knockdown of *Drosophila* Fgn did not increase MT levels but resulted in a higher proportion of the MTs being acetylated (Figure 3D).

### Fgn Depletion Decreases Ratio of Acetylated to Total Tubulin in the Axon

**Figure 4:**
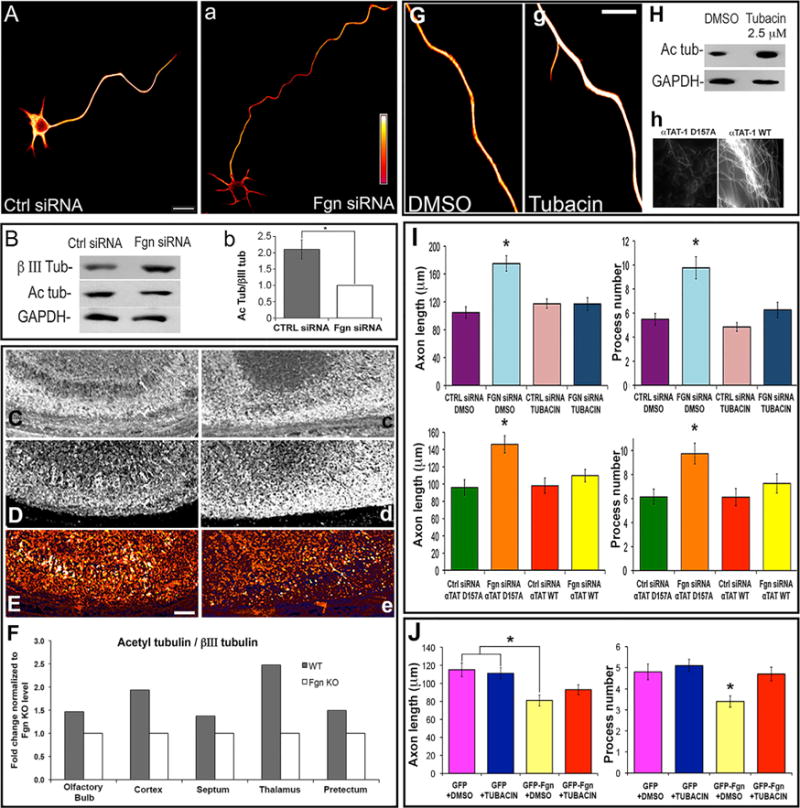
Fgn Is Sensitive to Acetylation Status of MTs in the Axon (A) Ratio images of acetylated tubulin to total βIII-tubulin displayed in fire-scale pseudo-color (white being the highest intensity and red being the lowest). Fgn siRNA neurons (right) display a decrease of acetylated to total tubulin ratio compared to Ctl siRNA. (B) Same result by western blot analysis with (right) quantification indicating that Ctl siRNA cultures have a higher ratio of acetylated to total tubulin compared to Fgn siRNA (fold change: Ctl siRNA, 2.1 ± 0.29; Fgn siRNA, 1; Student’s t test, p ≤ 0.05). (C–E) Brain immunohistochemistry of acetylated tubulin (C, left and right) and total βIII-tubulin (D, left and right) from E18.5 wild-type mouse and Fgn knockout/reporter mouse, respectively. Knockout brain has a decreased ratio of acetylated to total tubulin (E, right) compared to wild-type (E, left). (F) Quantification is shown as fold change normalized to the value of Fgn knockout ratio value as 1. Such difference was observed in all brain areas analyzed (the ratio value for the wild-type mouse are as follows: olfactory bulb, 2.2; cortex, 2.2; septum, 1.31; thalamus, 2.6; and pretectum, 1.9). (G) Ratio images in fire-scale pseudo-color of neurons treated with 2.5 μM tubacin or DMSO vehicle (Ctl) and immunostained for acetylated tubulin and total tubulin. The ratio of acetylated to total tubulin is higher with tubacin treatment. (H) (Top) Western blotting of parallel cultures also show an increase in acetylated tubulin. (Bottom) RFL-6 fibroblasts prepared for IF visualization of acetylated tubulin after overnight expression of an enzymatically inactive mutant of αTAT-1 (called αTAT-1 D157N) or the wild-type αTAT-1. Wild-type αTAT-1 shows higher staining intensity of acetylated MTs than the mutant. Similar results were obtained on neurons (data not shown). (I) Morphological quantification of experiments similar to those from Figure 2, except that MT acetylation was manipulated by either tubacin treatment or αTAT-1 overexpression. Ctl for the former and latter were DMSO vehicle and the αTAT-1 D157N, respectively. Neurons depleted of Fgn treated with vehicle (DMSO) have significantly longer axons (175 ± 11.04 μm; p ≤ 0.05, one-way ANOVA) and a higher number of minor processes (10 ± 0.9; 1 p ≤ 0.05, one-way ANOVA) compared to DMSO Ctl siRNA neurons (average axon length, 105 ± 8.29 μm; process number, 5 ± 0.49). With tubacin treatment, there were no morphological differences between Ctl (average axon length, 117 ± 6.80 μm; process number, 4.85 ± 0.37) and Fgn siRNA (average axon length, 117 ± 9.00 μm; process number, 6.26 ± 0.64; p > 0.05, one-way ANOVA for both measurements). Neurons depleted of Fgn expressing αTAT-1 D157N have longer axons (146 ± 9.78 μm; p ≤ 0.05, one-way ANOVA) and a higher number of minor processes (10 ± 0.86; p ≤ 0.05, one-way ANOVA) compared to αTAT-1 D157N Ctl siRNA neurons (average axon length, 96 ± 8.62 μm; process number, 6 ± 0.63). With αTAT-1 expression, there were no morphological differences between Ctl (average axon length, 98 ± 8.80 μm; process number, 6 ± 0.71) and Fgn siRNA (average axon length, 110 ± 7.21 μm; process number, 7 ± 0.79; p > 0.05, one-way ANOVA for both measurements). (J) The effects on morphology when GFP-Fgn-overexpressing neurons were treated with tubacin (2.5 μM). Neurons ectopically expressing GFP-Fgn and treated with vehicle (DMSO) show reduced minor process number (3.4 ± 0.27) and axon length (81 ± 6.04 μm) compared to neurons expressing GFP in the presence or absence of tubacin (minor process numbers, 5.1 ± 0.38 and 4.8 ± 0.38, respectively; axon lengths, 111 ± 6.32 and 115 ± 7.64, respectively; p < 0.05, one-way ANOVA). Scale bars represent 10 μm (A, left and G, right) and 100 μm (B, left). *p value ≤ 0.05; data are expressed as average ± SEM.

Cultures (Ctl-siRNA or Fgn-siRNA) double-labeled for IF visualization of total or acetylated tubulin in MTs revealed that total MT levels were elevated in the Fgn-depleted cultures, but acetylated MT levels remained roughly the same as in Ctl neurons (Figure 4A). This same reduction in the ratio of acetylated to total tubulin relative to Ctls was observed by western blotting of the cultured neurons (Figure 4B) and by immunohistochemistry on embryonic day (E)18.5 brain of the Fgn knockout/reporter mouse (Figures 4C–4F).

To investigate whether Fgn could be specifically targeting unacetylated tubulin, the same morphological experiments described earlier were conducted in the presence of tubacin, an inhibitor of HDAC6, the principal tubulin deacetylase in vertebrate cells (Sudo and Baas, 2010). Higher concentrations of tubacin can alter neuronal morphology, which is not unexpected given that the acetylation status of the MT affects how it interacts with a variety of different MT-related proteins. Chosen here was a lower concentration of the drug that had no statistically significant effect on axon length or process number, relative to DMSO (vehicle)-treated Ctls. However, even at this low dosage, there was an increase in tubulin acetylation as assessed by IF (ratio imaging of acetylated to total tubulin; Figure 4G) and western blotting (Figure 4H). Treatment of neurons with this dose of tubacin prevented Fgn siRNA from increasing axon length or minor process number relative to Ctl siRNA (Figure 4I, upper graphs), and it prevented overexpression of Fgn from reducing axon length and process number (Figure 4J).

Because acetyl transferases and deacetylases do not exclusively affect tubulin and may have other effects on MTs apart from their acetylation status, another approach was taken to further investigate. The chief tubulin acetyl transferase in vertebrate cells, namely α-TAT1 (Shida et al., 2010), was overexpressed. A mutant form of α-TAT1 (called α-TAT1 D157N) that cannot acetylate tubulin, but presumably can do anything else α-TAT1 may do, was used as a Ctl. Validation of the wild-type construct, but not the mutant, to increase MT acetylation is shown in Figure 4H. These constructs were transfected as GFP fusions into fetal cortical neurons just prior to re-plating neurons that had been transfected with Fgn siRNA 2 days prior. Then, 2 days later, the lengths of the axons and the number of minor processes from neurons expressing the constructs were quantified. Consistent with the tubacin results, both the increase in axon length and minor process number resulting from Fgn depletion were preserved in the presence of α-TAT1 D157N but were obliterated by the wild-type α-TAT1. Quantification of these results is shown in Figure 4I (lower graphs). Together, these and the tubacin results indicate that morphological changes resulting from increasing or decreasing the levels of Fgn depend on the presence of poorly acetylated MT domains.

## DISCUSSION

The present results indicate that vertebrate Fgn functions during development to tamp back the elongation of the labile domains of MTs so that they assemble in a regulated fashion. Overexpression of vertebrate Fgn does not result in obvious loss of MT mass from cells in which the labile domains are not expanding, but does suppress normal axonal elongation, a process that depends on the expansion of labile domains. Accordingly, depletion of vertebrate Fgn results in longer labile MT domains and longer axons. Changes in process number resulting from alterations in Fgn levels also can be explained this way. The situation is different in *Drosophila*, where the data suggest that Fgn functions as a traditional MT-severing protein, preferentially severing stable MT domains. Unlike flies, which only have one Fgn gene, vertebrates have two Fgn-like genes (Cox et al., 2000), which may have allowed Fgn itself to diverge. Vertebrate axons are much longer than *Drosophila* axons, which may be why vertebrates evolved machinery dissimilar from flies for regulating the growth capacity of the axon.

As for a mechanism, an initial idea was that vertebrate Fgn might co-hexamerize or co-dimerize with spastin and thereby act as a dominant-negative to spastin’s function. This idea came to mind because the phenotype of Fgn knockdown is similar to that of spastin overexpression, especially with regard to higher numbers of minor processes (Yu et al., 2008). Another idea is that the unique properties of vertebrate Fgn may result from the N-terminal domain of vertebrate Fgn absent from invertebrate orthologs acting in a regulatory fashion, similar to the situation with certain kinesins, where an inhibitory domain can fold over to suppress the activity of the functional domain (Cross and Scholey, 1999). However, neither of these ideas easily explains how Fgn preferentially severs labile domains of MTs. To this point, perhaps the amino acid substitutions in the Walker B domain weaken the ATPase activity such that vertebrate Fgn is only strong enough to break the lattice of the MT where it is weakest. This could result in a preference for the region of the MT toward its plus end, where much of the lattice has not yet fully closed. However, this would contrast with in vitro data suggesting that vertebrate Fgn has a preference for minus ends of MTs (Mukherjee et al., 2012). The present results support another possibility, namely that vertebrate Fgn targets labile domains of MTs through a preference for unacetylated tubulin. This idea is appealing because it would make Fgn the functional inverse of katanin, which targets stable domains through a preference for acetylated tubulin (Mao et al., 2014; Sudo and Baas, 2010).

Finally, there is the potential for therapeutic application. Inhibition of Fgn might be useful for treating neurodegenerative diseases as well as nerve injury, as a boost in labile MT mass may restore lost MT mass and/or enable a regenerating axon to grow with more vitality (Baas and Ahmad, 2013).

## EXPERIMENTAL PROCEDURES

### Fgn Conditional Knockdown Flies

Transgenic fly lines *UAS:CG3326*-*RNAi* (abbreviated as *UAS:Fgn*-*RNAi* here) were obtained from the Vienna *Drosophila* Resource Center (stock 24746). RNAi expression was restricted to neurons by using the *Elav^c155^*-*Gal4* driver (Robinow and White, 1991). Abdominal segment A3 from muscle 6/7 of third instar larvae was used for NMJ analysis. Immunohistochemistry was performed as previously described (Ghosh et al., 2014). Primary antibodies were as follows: anti-βIII-tubulin (BioLegend) specific for neurons and anti-acetylated tubulin (Sigma). Fluorescently conjugated α-HRP (1:125, Jackson ImmunoResearch Laboratories) was used to visualize neuronal membranes. NMJ images were obtained using an Olympus Fluoview 1000 laser-scanning confocal microscope.

### Fgn Knockout/Reporter Mouse

An Fgn knockout/reporter mouse (strain name: B6.129-Fign^tm1Frk/Frk^) in which a β-galactosidase reporter gene was inserted into the Fgn gene, replacing its catalytic domain (Cox et al., 2000), was purchased from Jackson Laboratory. Heterozygotes are fertile, while homozygotes die prenatally. X-gal staining of homozygote fetuses was conducted at E12.5. Brain immunohistochemistry for βIII-tubulin and acetylated tubulin was performed on homozygote fetuses at E18.5 (sagittal 20-μm sections) with the same antibodies used for the fly studies. Images were acquired with the confocal Leica TCS SP2 VIS/405.

### DNA Constructs and siRNA

The mEmerald-Fgn (termed GFP-Fgn, as mEmerald is a modified GFP) and FLAG-Fgn were generated from rat Fgn (NM_001106484.1). Fgn-GFP and EB3-GFP were provided by W. Frankel and N. Galjart, respectively. GFP-spastin was generated as described previously (Qiang et al., 2010).The α-TAT1 (pEF5B-FRT-GFP-α-TAT1) and α-TAT1 D157N (pEF5B-FRT-GFP-α-TAT1 [D157N]) were from Addgene. For siRNA experiments, cells were transfected with a pool of four rat Fgn sequences (Sigma-Aldrich, NCBI Reference Sequence (RefSeq): NM_001106484.1) or with a non-specific Ctl sequence (Ambion, AM4636), using the nucleofector device from Amaxa as previously described (Yu et al., 2008). The four sequences that comprise the pool also were used individually. The siRNA was used at 10 μM. DNA constructs were transfected by nucleofection into neurons or with Lipofectamine 2000 into fibroblasts.

### Cultured Rat Fetal Cortical Neurons

For MT quantification studies, cultures were pre-extracted in an MT-stabilizing buffer to release free tubulin as described previously, except that the detergent was 0.1% Triton X-100, prior to further processing for IF or western blotting (Baas and Black, 1990). Analyses were performed after 0 min, 30 min, or 4 hr in 2 μg/ml nocodazole or DMSO control (Baas and Black, 1990). For all other IF preparations, cells were fixed in 4% paraformaldehyde (PFA) and then post-extracted with 0.1% Triton. Images were acquired using identical exposure and other settings, thus allowing the comparisons between experimental groups. Ratio images of cultured neurons were obtained using the Zeiss Axiovert LSM 5 Pascal system with a HeNe laser microscope with no optical sectioning during imaging acquisition. Western blotting was performed by standard procedures, except that for the MT quantification experiments, the internal control was histone H3 level because GAPDH was lost during the pre-extraction step required to release free tubulin. Axon length was measured using Axiovision 4.6 software, considering as an axon any process longer than 50 μm. Minor processes were considered as any process longer than 6 μm but shorter than 50 μm. Developmental stages were defined as described in the Results. For Fgn depletion studies, more than 100 cells per condition per trial were analyzed. For overexpression studies, 30 cells per condition were analyzed in each trial. EB3 imaging was as previously described (Qiang et al., 2010). Three independent trials were performed for each analysis. For MT acetylation studies, tubacin was used at 2.5 μM and the α-TAT1 constructs were used as previously described (Shida et al., 2010). Three independent trials were performed in each study, and an average of at least 100 neurons per condition per trial were taken during the quantification. For some experiments, RFL-6, a rat fibroblast cell line, was used in addition to the primary cortical neurons.

### Statistical Analyses

All of the data analyses, statistical comparisons, and graphs were obtained by using SPSS 20 (IBM) and Excel (Microsoft). Data represent, if not otherwise specified, mean ± SD. Mean difference was considered to be significant at the 0.05 level (*p ≤ 0.05). Multiple group comparison was performed by one-way ANOVA followed by Bonferroni post hoc, and for pair comparison, Student’s t test.

### Institutional Review Board Approval

Rats and mice were used for the present studies under supervision and oversight from the Institutional Animal Care and Use Committees (IACUC) at Drexel University and Albert Einstein University College of Medicine.

## Supplementary Material

Supplemental figures S1 and S2

## Highlights

- Vertebrate fidgetin severs the labile domains of axonal microtubules
- Vertebrate fidgetin targets unacetylated tubulin
- Experimental manipulation of fidgetin leads to changes in axonal growth
