# Supplementary material for: Vertebrate Fidgetin Restrains Axonal Growth by Severing Labile Domains of Microtubules
Source: Cell Rep. Author manuscript; Available in PMC 2016 Apr 20. (PMC4837332; doi:10.1016/j.celrep.2015.08.017)
Supplement: Supplemental figures S1 and S2 [file NIHMS763086-supplement-Supplemental_figures_S1_and_S2.pdf]

Cell Reports

Supplemental Information

Vertebrate Fidgetin Restrains Axonal Growth by Severing Labile Domains of Microtubules

Lanfranco Leo, Wenqian Yu, Mitchell D'Rozario, Edward A. Waddell, Daniel R. Marenda, Michelle A. Baird, Michael W. Davidson, Bin Zhou, Bingro Wu, Lisa Baker, David J. Sharp, and Peter W. Baas

Vertebrate fidgetin restrains axonal growth by severing labile domains of microtubules

Leo et al.

Supplemental Figures

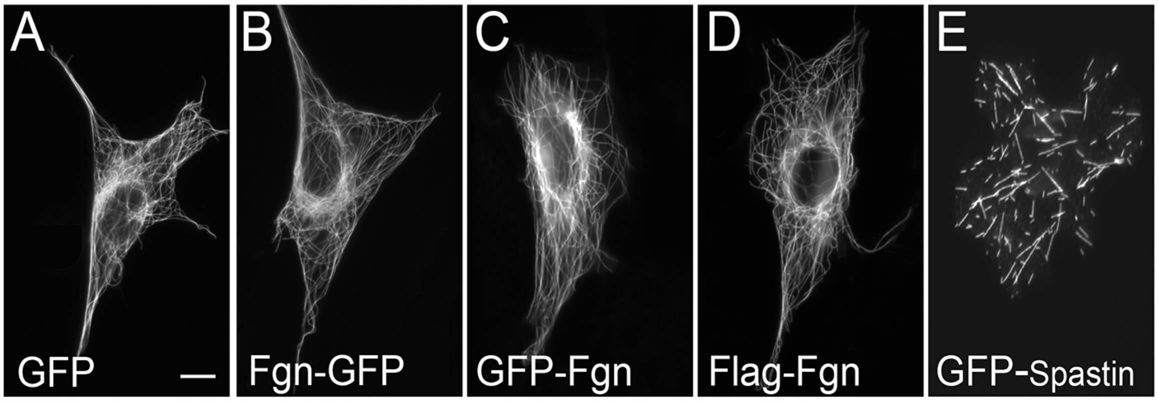

Supplemental Figure 1: Overexpression of Fgn in fibroblasts does not result in the appearance of short MT fragments, regardless of tag on Fgn construct. This figure is related to the Results sub-section entitled "Fgn expression in developing neurons" and Figure 2. A rat fibroblast cell line (RFL-6) was transfected with plasmids coding for: (B) Fgn-GFP (C-terminal GFP tagging); (**C**) GFP-Fgn (N-terminal GFP tagging); (**D**) FLAG-Fgn (N-terminal FLAG tagging); (**A**) empty vector; or (**E**) GFP-spastin. Cells were fixed and immunostained to reveal MTs. As expected, overexpressed spastin resulted in severing of the MT array into short MT fragments. This effect was not evident in the case of any of the Fgn constructs. Scale Bar, 10 μm.

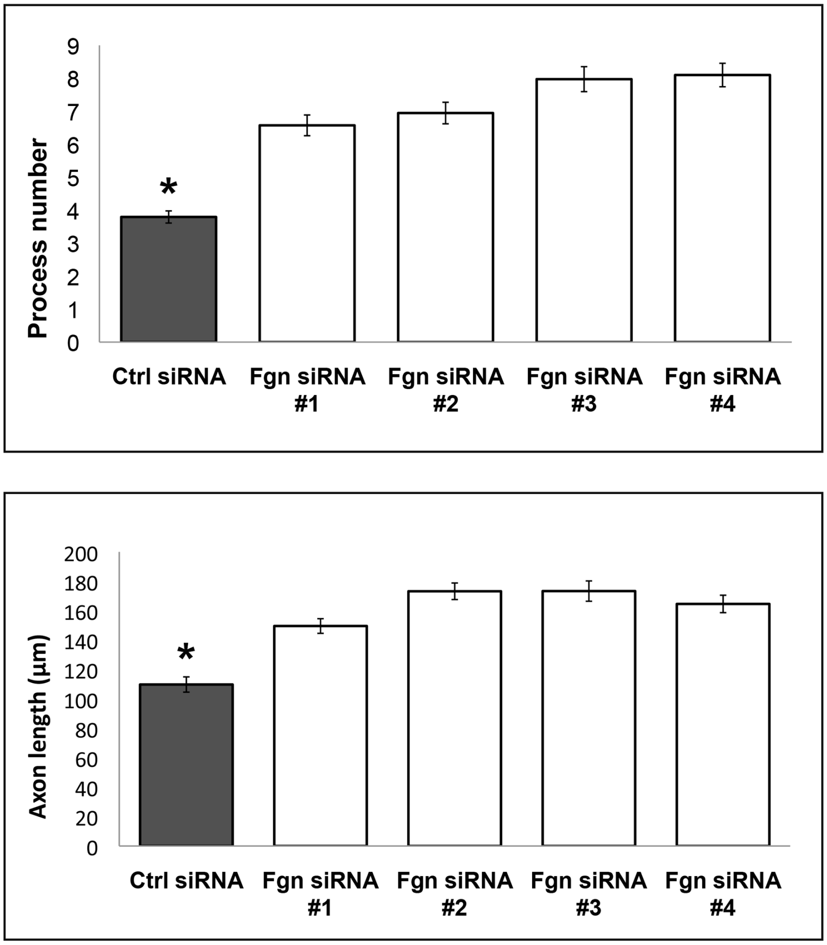

Supplemental Figure 2: Individual siRNA sequences produce the same phenotype as when they are pooled. This figure is related to the Results sub-section entitled "Effects of Fgn depletion on cultured vertebrate neurons" and Figure 2. Morphological analyses (axon length and minor process number) were performed on primary cortical neurons transfected with the Ctl siRNA or the same amount of each single sequence as the total amount of the pool used for studies shown in the main article. The neurons transfected with Ctl siRNA have statistically lower numbers of minor process and shorter axons (3.79±0.195 and 109.82±4.89 respectively, $p<0.05$ one way ANOVA) compared to the neurons transfected with a single Fgn siRNA sequence (Fgn siRNA seq.1: 6.57±0.33 and 149.61±6.10 respectively; siRNA seq.2: 6.94±0.35 and 173.23±6.10 respectively; siRNA seq.3: 7.97±0.46 and 173.35±8.05 respectively; siRNA seq.4: 8.10±0.38 and

164.62±6.91 respectively). The sign * indicates a statistical p value ≤ 0.05; all values are expressed as average ± S.E.M.
